# Multi-Electrode Array with a Planar Surface for Cell Patterning by Microprinting

**DOI:** 10.3390/s19245379

**Published:** 2019-12-05

**Authors:** Jan Slavík, Josef Skopalík, Ivo Provazník, Jaromír Hubálek

**Affiliations:** 1 Central European Institute of Technology, Brno University of Technology, Purkyňova 123, 616 00 Brno, Czech Republic; jaromir.hubalek@ceitec.vutbr.cz; 2 Department of Biomedical Engineering, Faculty of Electrical Engineering and Communication, Brno University of Technology, Technická 12, 612 00 Brno, Czech Republic; skopalik@feec.vutbr.cz (J.S.); provaznik@feec.vutbr.cz (I.P.); 3 Department of Physiology, Faculty of Medicine, Masaryk University, Kamenice 5, 625 00 Brno, Czech Republic; 4 Department of Microelectronics, Faculty of Electrical Engineering and Communication, Brno University of Technology, Technická3058/10, 616 00 Brno, Czech Republic

**Keywords:** multielectrode array, microelectrode array, sacrificial layer, peel-off, HL-1 cells, microprinting, antifouling agent

## Abstract

Multielectrode arrays (MEAs) are devices for non-invasive electrophysiological measurements of cell populations. This paper describes a novel fabrication method of MEAs with a fully planar surface. The surface of the insulation layer and the surface of the electrodes were on one plane; we named this device the planar MEA (pMEA). The main advantage of the pMEA is that it allows uniform contact between the pMEA surface and a substrate for positioning of microfluidic channels or microprinting of a cell adhesive layer. The fabrication of the pMEA is based on a low adhesive Au sacrificial peel-off layer. In divergence from conventional MEAs with recessed electrodes, the electrodes of the pMEA lead across the sloped edge of the insulation layer. To make this, the profile of the edge of the insulation layer was measured and the impedance of the planar electrodes was characterized. The impedance of the pMEA was comparable with the impedance of conventional MEA electrodes. The pMEA was tested for patterning HL-1 cells with a combination of imprinting fibronectin and coating by antifouling poly (l-lysine)-graft-poly(ethylene glycol) (PLL-g-PEG). The HL-1 cells remained patterned even at full confluency and presented spontaneous and synchronous beating activity.

## 1. Introduction

Multielectrode arrays (MEAs) are devices containing microelectrodes on the surface that are cultivated as excitable cells for in vitro experiments or positioned on tissue for in vivo experiments. Electrodes measure extracellular field potentials generated by the action potential and can also stimulate cells. MEAs provide non-invasive, long term measurements to study the electrophysiology of cell populations in cardiology and neuroscience. Electrodes measure field potentials in their surroundings; therefore, smaller electrodes can localize the origin of the field potential more precisely, while bigger electrodes are more suitable for measuring field potentials from a cell’s monolayer [1,2].

MEAs have a specific application in combination with other contact techniques. A microfluidic culture platform for neuron culturing can be placed and aligned on an MEA surface where the microchannels allow neurites to grow and make defined interconnections. By specific design, it is possible to distinguish patterning axons from dendrites [3,4,5,6,7]. Cells that are seeded on MEA can be patterned by the microprinting method. Various coating agents and stamp dimensions have been applied to form well defined neural networks [8,9,10,11,12]. Cells that are not sensitive to the coating agent could be patterned by a combination of microprinting the coating agent and absorption of the antifouling agent [13,14].

All of the afore-mentioned techniques would be advantageous to MEAs with a fully planar surface. Polydimethylsiloxane (PDMS) structures must level down into the hole of the insulation layer onto the electrode surface; this creates defects on non-contacted surfaces. For instance, microprinting with a PDMS stamp on an MEA would leave unpatterned spaces on the edges of the insulation layer of the electrodes, which would then be available for subsequent coating by the antifouling agent. The severity of the defect depends on the size of electrodes and the thickness of the insulation layer. If the electrodes and insulation layer are on one plane, the electrode’s surface is then fully accessible without defects to the imprinting transfer process of the microprinting method and placing culturing platforms or microfluidic channels. MEAs with a fully planar surface would also avoid trapping air pockets during MEA positioning for in vivo applications [15].

We present a new fabrication process of MEAs with planar surfaces (pMEAs), based on a sacrificial Au peel-off layer. We characterized electrodes and tested pMEA for patterning excitable cells with the microprinting method. For our experiment, we used HL-1 cells, which were derived from AT-1 mouse atrial cardiomyocytes. HL-1 cells retain some cardiac cell properties, including the ability to contract. The HL-1 cells can be passaged while still keeping their properties [16,17]. HL-1 cells have already been used as a model to study electrophysiology on MEAs [18,19,20].

## 2. Materials and Methods

### 2.1. The Fabrication of MEA

**Figure 1:**
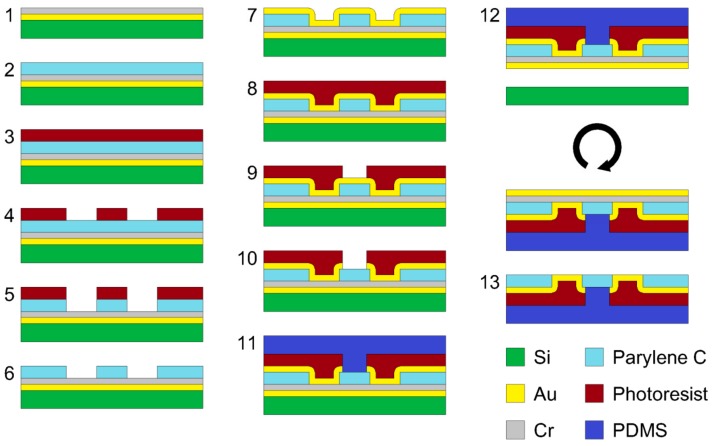
Fabrication steps of the planar multielectrode array (pMEA).

The fabrication steps of the pMEA are shown in Figure 1. Step 1: The silicon wafer was sputtered by a magnetron sputtering 150 nm of Au at a deposition rate of 0.13 nm/s and 150 nm of Cr at a deposition rate of 0.12 nm/s. Step 2: The substrate was coated by a 3.5 µm parylene C layer. Step 3: The substrate was spin coated by AZ9260 positive photoresist (MicroChemicals GmbH, Ulm, Germany) at 5000 RPM for 60 s to obtain a 6 µm thick photoresist. The photoresist was soft baked on a hotplate at 110 °C for 165 s. Step 4: The design of the holes for the electrodes was exposed by direct write laser (DWL) with a power of 10 mW. The AZ9260 photoresist was developed in AZ^®®^ 400 K developer DI water diluted to 1:2 for 60 s, and then, the substrate was rinsed in DI water and dried with N_2_ gas. Step 5: Parylene C was etched by reactive ion etching (RIE) fluorine at a pressure of 200 mTorr, with a power of 100 W, O_2_ = 50 sccm for 35 min. Step 6: AZ9260 photoresist was removed by acetone, and then, the substrate was rinsed in DI water and dried with N_2_ gas. Step 7: The substrate was sputtered by a magnetron sputtering 150 nm of Au at a deposition rate of 0.04 nm/s. Step 8: the substrate was spin coated by AR-N-4340 negative photoresist (Allresist GmbH, Strausberg, Germany) at 2000 RPM for 60 s to obtain a 2.1 µm thick photoresist. The photoresist was soft baked on a hotplate at 90 °C for 60 s. Step 9: The design of electrodes was exposed by DWL with a power of 20 mW. The AR-N-4340 photoresist was baked at 95 °C for 120 s to crosslink it, and then, it was developed in AR 300-475 developer for 60 s. The substrate was then rinsed in deionized (DI) water and dried with N_2_ gas. Step 10: The photoresist residue was removed by RIE fluorine at a pressure of 200 mTorr, with a power of 100 W, O_2_ = 50 sccm for 20 s. Then, the Au was etched by RIE fluorine at a pressure of 50 mTorr and a power of 200 W, Ar = 50 sccm for 15 min. Step 11: The PDMS was prepared by mixing the base polymer and curing agent at a ratio of 10:1. The solution was then mixed, degassed, and poured onto the substrate. The amount of poured PDMS defined the final thickness of the MEA, which was 1 mm. The PDMS was left to level up and then cured on a hotplate at 100 °C for 5 min. Step 12: The substrate was diced by a laser dicer, and the MEA was peeled off from the silicon. Step 13: The Au sacrificial peel-off layer was etched by gold etchant (Sigma Aldrich), and the Cr layer was etched by chromium etchant (16% of (NH_4_)_2_ Ce(NO_3_)_6_ with 6% HClO_4_ in DI water, Sigma Aldrich, St. Louis, MO, USA).

**Figure 2:**
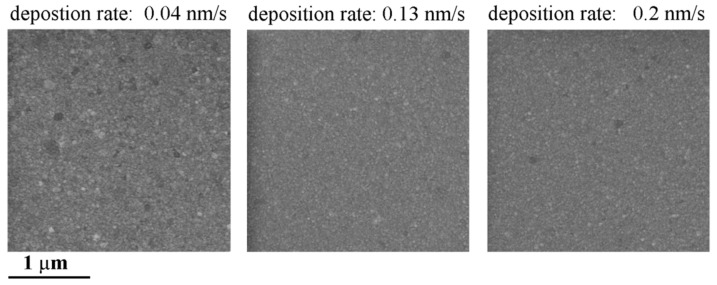
SEM pictures of Au sacrificial layers deposited at different deposition rates.

The first layer of Au sputtered on silicon served as the sacrificial peel-off layer. Adhesion between the Au layer and silicon substrate was the lowest in the whole structure, so that the pMEA could simply be peeled off from the silicon. The adhesion of the Au layer depended on the sputtering deposition rate. If the deposition rate was too slow, e.g., at a rate of 0.04 nm/s, the Au would adhere strongly on the silicon surface. Thus, the weakest adhesion would be between the PDMS and the structures under it, and thus, the PDMS would peel off instead of the Au. On the other hand, if the Au deposition rate was too high, e.g., 0.2 nm/s, the Au layer adhesion would fail during the sample heating in the process of the RIE of the Au electrodes. In this case, the Au sacrificial peel-off layer would rise as bubbles appeared, and the surface of the fabricated pMEA would be dimpled. A well peeled off layer without any defects had a deposition rate of around 0.13 nm/s. Furthermore, if the Au layer was too thin, the pMEA tended to have a wrinkled surface after peeling off. The SEM images of the Au sacrificial layers are shown in Figure 2. The surface roughness of Au sacrificial layers was measured by atomic force microscopy (Bruker, Billerica, MA, USA). The grains sizes were smaller for a lower deposition rate, while the surface roughness decreased with increasing deposition rate. At a deposition rate of 0.04 nm/s, the surface roughness was 1.47 ± 0.17 nm; at a deposition rate of 0.13 nm/s, the surface roughness was 1.23 ± 0.19 nm; and at deposition rate of 0.2 nm/s, the surface roughness was 1.09 ± 0.16 nm.

At a high growth rate, the sputtered atoms were still moving on the substrate while new particles were sputtered. This caused stress in the interfacial region between the Au layer and the substrate, and if the stress was too high, adhesion of the Au layer failed [21]. A highly stressed Au sacrificial layer could easily be damaged, even by tweezers. Finally, the Cr layer on top of the Au sacrificial layer served only as a protective layer for the Au electrodes that were not to be etched by the gold etchant. 

The Au conductive layer was deposited without an adhesion layer, and therefore, it could be more easily mechanically damaged. To increase the durability of the pMEA, the Au conductive layer was sputtered at a slow deposition rate. Furthermore, all edges of the Au conductive layer should be under an insulation layer. This included pads, reference, and measuring electrodes. Then, the pMEA would withstand contact with a PDMS stamp without damage.

**Figure 3:**
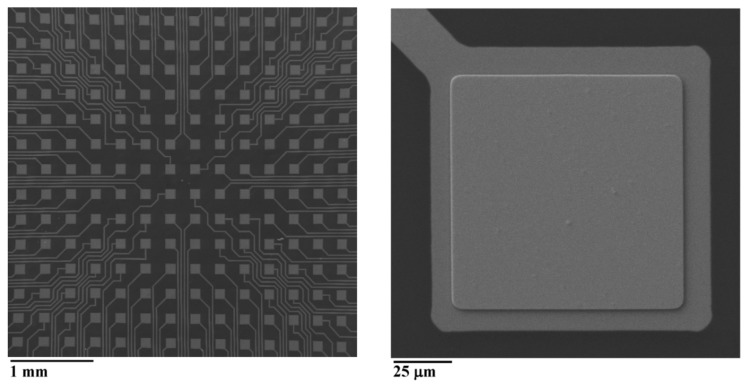
On the left, the SEM picture of the pMEA. On the right, the SEM picture of the planar electrode.

The final pMEA had PDMS as a substrate and 254 Au electrodes with dimensions of 100 µm × 100 µm. The distance between the centers of the electrodes was 300 µm with a 3.5 µm thick parylene C insulation layer. An SEM picture of the fabricated planar electrode is shown in Figure 3.

The flexible PDMS (hardness Shore A 48 [22]) can be substituted by solid epoxy resin. A non-transparent MC35.1–W21 epoxy resin (ELCHEMCo, Zruč nad Sázavou, Czech Republic) (hardness Shore D 90) was successfully tested as the substitute.

### 2.2. Characterization of Planar Electrodes

The profile of the sloped edge of the etched parylene C insulation layer was measured by a mechanical profilometer (DektakXT, Bruker, Billerica, MA, USA). The sloped edge of the insulation layer was measured from the bottom up, to ensure that the tip of the stylus scanned the surface and not the stylus edge.

The impedance of electrodes was measured by Autolab (PGSTAT204, Metrohm, Herisau, Switzerland). As an electrolyte, a 0.9% NaCl solution was used. The electrode of the pMEA was the working electrode; the Ag/AgCl was the reference electrode; and the Pt wire was the counter electrode. A 50 mV sine wave was applied to the electrode with a frequency range of 0.1 Hz to 100 kHz. The impedances of five different electrodes were measured, and statistical analysis was performed in R software.

### 2.3. The Fabrication of the PDMS Stamp and Microprinting Method

The master for the PDMS stamp was fabricated from a 1.5 inch silicon wafer. The silicon wafer was firstly spin coated by AZ9260 photoresist at 2400 RPM for 60 s and soft baked at 110 °C for 80 s. The silicon wafer was then spin coated a second time by AZ9260 photoresist at 2100 RPM for 60 s and soft baked at 110 °C for 160 s. The final thickness of the AZ9260 photoresist was 24 µm. The design of lines on the AZ9260 photoresist was exposed by a DWL with a power of 20 mW. The AZ9260 photoresist was developed in an AZ^®®^400K developer diluted with DI water 1:2 for 60 s, and then, the wafer was rinsed in DI water and dried with N_2_ gas. The master was treated by hexamethyldisilazane vapors in a chamber at 100 °C for 10 min and then left to cool down.

The PDMS was prepared by mixing the base polymer and curing agent at a ratio of 10:1, and the solution was mixed, degassed, and poured onto the master. The PDMS was left to level up and was cured on a hotplate at 100 °C for 5 min. Then, PDMS was then peeled off. The PDMS stamp had raised features 24 µm tall and 180 µm wide, and the distance between the raised features was 120 µm.

**Figure 4:**
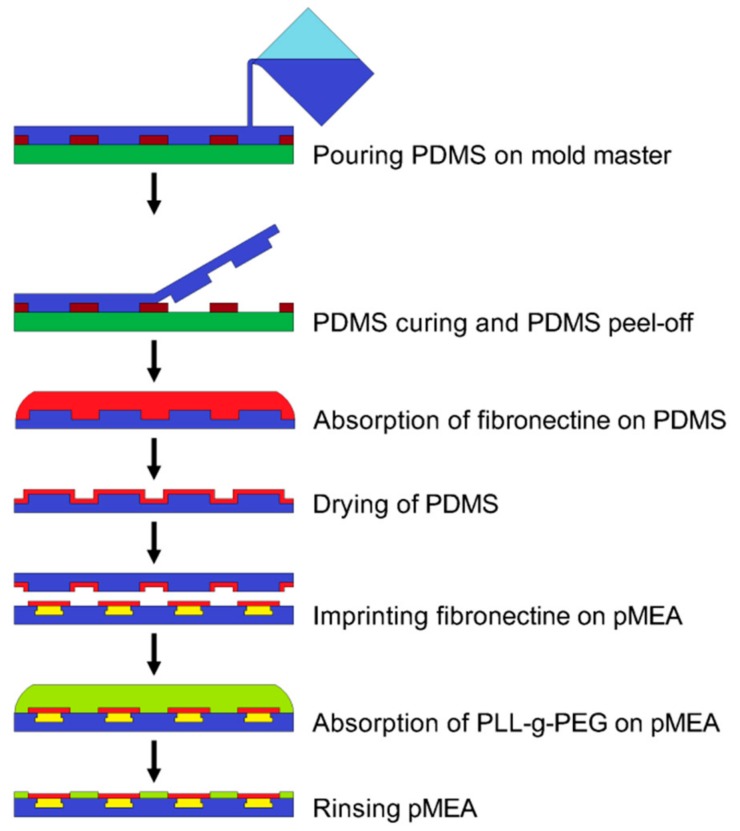
Scheme of the microprinting of fibronectin and coating by PEG-g-PLL.

A drop of 50 μg/m: fibronectin in 10 mM phosphate buffered saline (PBS) with a pH of 8.5 was added to the PDMS stamp surface. The fibronectin was adsorbed onto the PDMS stamp surface for 1 h, and then, the PDMS stamp was rinsed in DI water and gently dried with N_2_ gas. The pMEA surface was treated with oxygen plasma at O_2_ = 50 sccm with a power of 40 W for 20 s to enhance the PLL-g-PEG coating. The PDMS stamp was aligned according to the pMEA electrodes with an optical prism and put into contact with the pMEA surface for 60 s. The PLL (20)-g-[3,5]-PEG (2) powder (SurfaceSolutions, Dübendorf, Switzerland) was dissolved in a 10 mM HEPES buffer with a pH of 7.4. The PLL-g-PEG solution was prepared in a concentration of 0.1 mg/mL. The pMEA with patterned fibronectin was coated by with PLL-g-PEG solution for 30 min. The PLL-g-PEG solution was removed, and the pMEA was rinsed twice by PBS (Ca^2+^ free and Mg^2+^ free). The fabrication of the PDMS stamp, the microprinting of the fibronectin, and the coating of the pMEA surface by antifouling PLL-g-PEG is shown in Figure 4.

### 2.4. HL-1 Cell Culturing, Measurements of Action Potentials, Staining, and Visualization

HL-1 cells (cardiac myocytes from transgenic mice) were obtained from Sigma Aldrich. HL-1 cells were cultured on the plastic bottom of a T25 flask in Claycomb Medium, supplemented with 10% fetal bovine serum, 100 U/mL penicillin, 100 mg/mL streptomycin, 0.3 mM ascorbic acid, 10 mM norepinephrine, and 2 mM L-glutamine (all ingredients from Sigma-Aldrich), at 37 °C in a humid atmosphere of 5% CO_2_. The Claycomb Medium was renewed every second day. Cells (split by trypsin) at full confluency from Passages 15–20 were used for seeding the pMEA at a density of 75,000 cells/cm^2^.

After 24 h, the pMEA seeded with HL-1 cells was connected to a commercial measurement system (MEA2100-Systems, Multichannel Systems, Reutlingen, Germany). Measurements were taken in the room atmosphere while the pMEA was heated to 37 °C by an integrated heating element of MEA2100. The spontaneous activity of HL-1 cells was measured by a commercial program (Multichannel Experimenter, Multichannel Systems, Reutlingen, Germany) and analyzed by a commercial program (Multichannel Analyzer, Multichannel Systems, Reutlingen, Germany).

After the measurements, the HL-1 cells were fixed onto the pMEA with 4% paraformaldehyde for 30 min, and the membranes were permeabilized at 0.001% triton X-100 for 5 min. They were then stained with actin green antibodies (AA448, Invitrogen, Thermo Fisher Scientific, Waltham, MA, USA) for 5 min, washed with PBS, and visualized with a fluorescence microscope (ZEISS, Axio Imager 2, Jena, Germany).

pMEAs without fixed cells could be cleaned by a cleaning procedure recommended for commercially manufactured MEAs. The procedure consisted of removing any cultivation medium, rinsing the pMEA with distilled water, placing the pMEA in a 1% solution of Terg-A-Zyme overnight at room temperature, and rinsing the pMEA with distilled water.

## 3. Results and Discussion

### 3.1. The Profile of the Sloped Edge of the Insulation Layer and the Impedance of the Planar Electrodes

Impedance is the basic parameter for the characterization of MEAs. Bigger electrodes have lower impedance and thus lower noise. However, bigger electrodes record less localized information, which is more suitable for the measurement of cell populations rather than individual cells.

For a general comparison, MEA impedance was measured at 1 kHz for a 50 mV sine. As the electrolyte, 0.9% NaCl solution was used. The expected value for electrodes with an unmodified surface is hundreds of kΩ. However, the conductive layer of the pMEA was led across the sloped edge of the insulating layer, where the conductive layer was thinner. This reduced the conductivity and increased impedance. Therefore, the RIE of parylene C was done at high pressure (200 mTorr) to achieve a high under-etched edge of the insulation layer, so creating a flattened profile.

**Figure 5:**
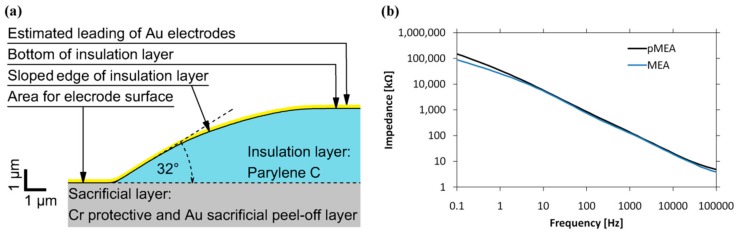
(**a**) The profile of the sloped edge of the insulation layer; (**b**) the impedance of the pMEA and the MEA with recessed electrodes.

The profile of the sloped edge of the insulation layer was measured by a mechanical profilometer following Fabrication Step 6 from Figure 1. The profile was not linear. The sharpest slope in the profile was at the bottom edge of the insulation layer, where the angle measured 32°. The whole profile is shown in Figure 5a.

To investigate how the sloped edge affected the impedance of the pMEA, a conventional MEA with recessed electrodes was fabricated for the impedance comparison. The MEA with recessed electrodes was fabricated by a common microfabrication process. Briefly, the substrate was glass; the electrodes were 150 nm of Au with 10 nm of Ti as an adhesion layer patterned by wet etching; and 3.5 µm of parylene C was used as the insulation layer. The electrodes had dimensions of 100 µm × 100 µm.

**Figure 6:**
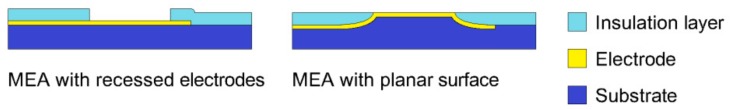
Difference of shape of the MEA with recessed electrodes and the MEA with a planar surface.

The impedance of the pMEA and MEA is shown in Figure 5b. The impedance at 1 kHz for the pMEA was 136 kΩ, and for the MEA with recessed electrodes, the impedance at 1 kHz was 120 kΩ. The impedance of the pMEA was similar to the impedance of the MEA with recessed electrodes. Leading electrodes across the flattened sloped edge of the insulation layer did not significantly affect the impedance of electrodes. MEA and pMEA impedance curves were recorded in five independent measurements. The impedances were transformed logarithmically for visual comparison (see Figure 5b). We applied the Kolmogorov–Smirnov test and found the impedances to be statistically not different (the impedance values were not from different distributions, *p* = 1000). However, the correlation coefficient between MEA and pMEA was 1000 (*p* < 2 × 10^−16^). The difference in shape of the MEA with recessed electrodes and the MEA with a planar surface is shown in Figure 6.

### 3.2. Cell Patterning on pMEA by Microprinting

Microprinting with a PDMS stamp is a well described method [23,24]. The height of the raised features (24 µm) was sufficient to avoid roof collapse of the PDMS stamp. The pressure applied to the PDMS stamp was 25 kPa. However, the pressure applied on the PDMS stamp and the length (time) of the pressure were not critical parameters for successful imprinting of the fibronectin. According to our preliminary experiments (results not shown), HL-1 cells grew well on various surfaces (silicon, glass, PDMS, parylene C), even without coating. Patterning HL-1 cells just by imprinting of fibronectin was not possible. Therefore, for patterning, we used a combination of the microprinting method with an antifouling agent coating. The antifouling agent we used was PEG. PEG is a polynonionic polymer of a polyether compound that blocks biological recognition as cell adhesion. To allow adherence to a surface, the PEG is synthesized as a copolymer with PLL (together called PLL-g-PEG). The structure of the copolymer is a comb, with a positively charged PLL backbone that adheres to a negatively charged surface while the chains of PEG are exposed to the surroundings. Therefore, the cell cannot adhere to a surface coated by PLL-g-PEG [25]. A combination of imprinting fibronectin with a PDMS stamp and PLL-g-PEG coating to pattern cells was already described [14], as have other similar methods [26]. To enhance PLL-g-PEG adhesion, the surface can be treated with oxygen plasma, but only for a short time, since oxygen plasma also etches parylene C. Parylene C treated by plasma will remain oxidized over time [27,28], unlike other commonly used materials such as glass or PDMS.

### 3.3. A Measure of HL-1 Cells’ Action Potential on the pMEA

**Figure 7:**
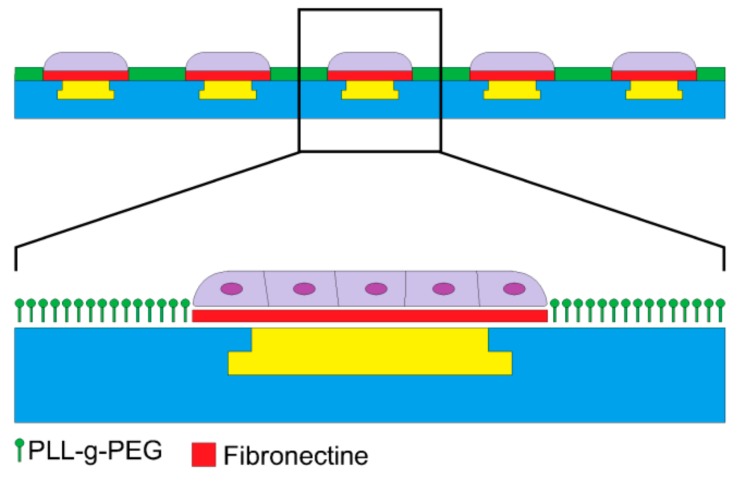
The scheme of HL-1 cells patterned on planar electrodes.

**Figure 8:**
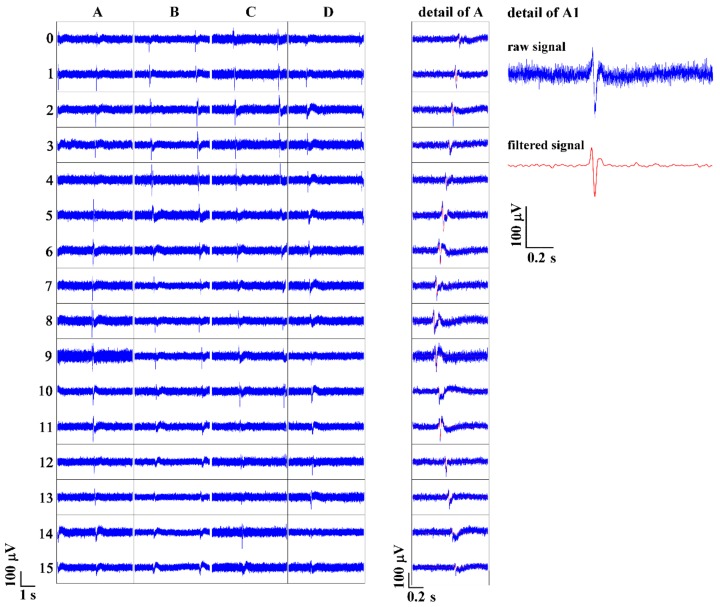
On the left, HL-1 cells AP propagation over the electrodes of the pMEA. The HL-1 grows in separate lines with their individual beating activity. In the middle, the detail of AP propagation over one column. The cell with the highest intrinsic frequency was between Electrodes A8 and A9. On the right is shown the raw signal and the filtered signal from Electrode A1.

The scheme of expected alignment of HL-1 cells on the pMEA is shown in Figure 7. After 24 h, fully confluent HL-1 cells already formed gap junctions while they were still patterned in lines. The cells in lines presented their individual spontaneous and synchronous beating activity. Each line had an individual beat rate corresponding to an individual pace marker cell with the highest intrinsic frequency (Figure 8). For the raw signal, the average HL-1 peak from the upper amplitude to the lower amplitude was 160 ± 40 µV, and the noise was 11.3 ± 0.7 µV calculated as 2∙MAD (mean absolute deviation), while the signal-to-noise ratio (SNR) was 14.15. The raw noise calculated as RMS (root mean square) was 18 ± 1.2 µV, and SNR was 8.9. The raw signal was filtered by a low pass filter of 50 Hz and a high pass filter of 5 Hz. For the filtered signal, the average HL-1 peak was 101 ± 30 µV, and the noise was 1.6 ± 0.1 µV (2∙MAD), while the signal-to-noise ratio increased to 63.1. The filtered noise calculated as RMS (root mean square) was 3.2 ± 0.1 µV, and SNR was 31.6. Both MAD and RMS could be used for the determination of noise variability, while MAD suppresses the peaks in the signal more in comparison to RMS.

**Figure 9:**
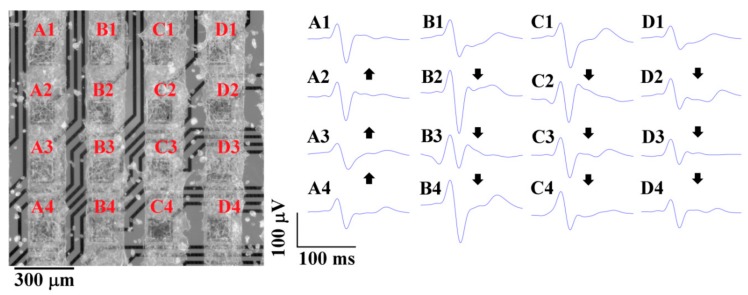
On the left, a fluorescent image of the planar electrodes with patterned HL-1 cells overlapped by a partially transparent corresponding bright field image to highlight the electrodes’ positions. On the right, the measured and averaged spontaneous AP of the HL-1 cells. The direction of AP propagation is marked with arrows. The velocities of AP propagation were: A, 6.95 mm/s; B, 6.12 mm/s; C, 7.4 mm/s; D, 7.69 mm/s.

After measurement, HL-1 cells were fixed, stained, visualized, and measured, and the electrical activity was assigned in the figure to the corresponding electrodes (Figure 9). The propagation velocities of action potentials (APs) were defined as the distance between the detected thresholds of peaks. The direction of AP propagation was also individual for HL-1 cells patterned in lines. We analyzed AP propagation velocities in four clearly defined HL-1 cell columns, marked as A, B, C, and D in Figure 8. The velocities of the AP propagation were: A, 6.95 mm/s; B, 6.12 mm/s; C, 7.4 mm/s; D, 7.69 mm/s. In other studies, the reported AP propagation velocities were comparable: 4.6 mm/s [29] and 5−40 mm/s [30]. The study [17] presented a significantly higher velocity of 47–320 mm/s.

## 4. Conclusions

In this paper, we described a new method of fabricating MEA with a fully planar surface (pMEA). The surface of the insulation layer and electrodes was on one plane. The fabrication process was based on an Au sacrificial peel-off layer. The edge profile of planar electrodes was characterized, and the planar electrode impedance was measured and compared with the impedance of a conventional recessed electrode. On the pMEA surface, the HL-1 cells were patterned by a combination of the microprinting method and antifouling agent coating. The HL-1 cells’ electrical activity was measured and analyzed, and the HL-1 cells were visualized on the pMEA surface. A combination of real-time imaging of the cell culture on the pMEA and real-time visualization of the electrical activity of the MEA2100 on the same computer screen could offer an effective tool for precise studies of excitation conduction. Further, it allows for the study of factors that influence the excitation rate in cell cultures and the modelling of tissue adhered to the pMEA. The advantages of a plane surface are precision and the avoidance of defects on the MEA with various contact methods such as microprinting, positioning culture, or microfluidic platforms on the MEA surface and the avoidance of the formation of air pockets during positioning of the MEA for in vivo applications.
